# Sleep, Aging, and Cellular Health: Aged-Related Changes in Sleep and Protein Homeostasis Converge in Neurodegenerative Diseases

**DOI:** 10.3389/fnagi.2019.00140

**Published:** 2019-06-11

**Authors:** Jennifer M. Hafycz, Nirinjini N. Naidoo

**Affiliations:** Center for Sleep and Circadian Neurobiology, Perelman School of Medicine, University of Pennsylvania, Pennsylvania, PA, United States

**Keywords:** sleep, aging, proteostasis cell stress and aging, neurodegenarative disease, unfolded protein response

## Abstract

Many neurodegenerative diseases manifest in an overall aged population, the pathology of which is hallmarked by abnormal protein aggregation. It is known that across aging, sleep quality becomes less efficient and protein homeostatic regulatory mechanisms deteriorate. There is a known relationship between extended wakefulness and poorly consolidated sleep and an increase in cellular stress. In an aged population, when sleep is chronically poor, and proteostatic regulatory mechanisms are less efficient, the cell is inundated with misfolded proteins and suffers a collapse in homeostasis. In this review article, we explore the interplay between aging, sleep quality, and proteostasis and how these processes are implicated in the development and progression of neurodegenerative diseases like Alzheimer’s disease (AD). We also present data suggesting that reducing cellular stress and improving proteostasis and sleep quality could serve as potential therapeutic solutions for the prevention or delay in the progression of these diseases.

## Introduction

Sleep is found in all animals that have been studied to date. It is a universal and crucial aspect of overall health. While the specific functions of sleep are still actively researched, some benefits of sleep, including memory consolidation (Stickgold and Walker, 2007; Diekelmann and Born, 2010) and management of cellular processes (Benington and Heller, 1995; Cirelli et al., 2004), have been demonstrated. However, it is known that sleep quality decreases with age (Manderville and Wetmore, 2017) and poor sleep is often a symptom of many aged-related neurodegenerative diseases, such as Alzheimer’s disease (AD; Ju et al., 2013). This begs the question to what extent is sleep causally related to the progression of aging and the symptoms of neurodegeneration. Perhaps the most effective way to examine this question is to investigate cellular health at the molecular level. Most neurodegenerative diseases display protein aggregation as a main feature of the disease, suggesting that proteostatic dysfunction could be causally related to the development and progression of these diseases. Proteostasis, or protein homeostasis, is maintained by quality control systems and signal transduction pathways such as the unfolded protein response (UPR; Braakman and Hebert, 2013). The UPR is initiated in the endoplasmic reticulum (ER), which is a major site of protein folding and processing. The ER is responsible for ensuring proper protein folding of all secretory and membrane proteins, through the use of other chaperone proteins. Protein misfolding due to changes in the ER environment such as redox, energy, pH and calcium perturbations activate the UPR (Kaufman, 2002; Ron, 2002). Activation of the UPR is an adaptive response and works in the short term to restore proteostasis (Koga et al., 2011). Importantly, as we age, the protein regulatory mechanisms of the cell deteriorate, as does sleep quality (Koga et al., 2011). This review will explore the interplay of sleep quality and protein homeostatic changes across aging, with a focus on the UPR, and the implications that these changes have for the development, progression, and treatment of neurodegenerative diseases. The goal of this is review is to present a cellular perspective on sleep and aging in terms of neurodegenerative diseases that can have broad impacts on the overall health of the individual across the normal aging process, and can serve as a potential therapeutic target for these diseases.

## Sleep and Proteostasis

Sleep is a ubiquitous physiological process. The current theory for the physiological regulation of sleep is the two-process model of sleep (Borbély, 1982). This model suggests that there is a circadian component to regulate the timing of sleep across the 24-h day, as well as a homeostatic process that regulates sleep based on the homeostatic regulatory processes of the cell (Borbély, 1982). The circadian process is regulated by light across the 24-h day as well as the cycling of proteins such as melatonin (Cassone, 1990; Brown, 1994). For the purposes of this review, however, we will focus on the homeostatic process, which regulates sleep on a cellular/molecular level based on cellular demands. This hypothesis posits that there is a buildup of molecules that correlate with the duration of wakefulness. Extending wakefulness, therefore, results in an accumulation of molecules and proteins commensurate with the prior duration of wakefulness.

The waking cell is incredibly active transcribing DNA and translating proteins that are necessary to maintain wake function. As mentioned previously, the ER is the site of secretory and membrane protein synthesis in the cell, with the neuron being no exception. The ER of neurons extends through the entire axon and cell body of the neuron (Berridge, 2002; Ramírez and Couve, 2011). The vastness of this organelle, relative to the size of the neuron itself, indicates that protein synthesis is especially critical for neural function. Newly synthesized proteins are located in the ER lumen, which contains molecular chaperones that bind to the newly formed proteins in order to assist them in achieving their appropriate conformation. Once proteins are properly folded, they are transferred out of the ER to serve their cellular function. If the proteins cannot be properly folded, they are targeted to the proteasome for degradation to prevent aggregation (Ellgaard et al., 1999; Ellgaard and Helenius, 2001). Despite quality checkpoints and the presence of molecular chaperones, some proteins will be misfolded and will accumulate in the lumen of the ER.

There are instances when protein accumulation in the ER is too overwhelming for chaperone proteins alone. This state, once reached, is referred to as ER stress, which initiates a cascade of cellular signaling collectively referred to as the UPR, to cope with the aggregated proteins in the lumen of the ER (Berridge, 2002). This UPR has many downstream consequences for the cell and affects many important cellular mechanisms, including metabolic pathways (Bravo et al., 2013). The UPR activates three main types of protective responses: up-regulation of chaperone proteins such as BiP [also known as glucose regulated protein 78 (GRP78) or Hspa5], down-regulation of protein translation, and increasing degradation of misfolded proteins. When there is extended stress on the ER and these three mechanisms of the UPR are not sufficient to relieve that stress, apoptotic pathways are activated, ultimately leading to cell death (see reviews by Ron and Walter, 2007); and (Szegezdi et al., 2006). Interestingly, several microarray studies have shown that protein translation factors and ER resident factors such as BiP that are involved in these pathways of the UPR are increased with prolonged wakefulness, or sleep deprivation (Cirelli and Tononi, 2000; Cirelli et al., 2004; Naidoo et al., 2005; Mackiewicz et al., 2007; Naidoo et al., 2007). These observations suggest that protein homeostatic mechanisms could be linked to sleep/wake patterns.

There are three ER transmembrane proteins that serve as the “stress detectors” of the cell; PKR-like endoplasmic reticulum kinase (PERK), inositol requiring enzyme 1 (IRE1), and activating transcription factor 6 (ATF6; Zhang and Kaufman, 2004; Ron and Walter, 2007). BiP is bound to these three receptors under normal cellular conditions. When misfolded proteins are present, BiP dissociates from these receptors, thereby activating their signaling pathways, and binds to the unfolded proteins. Activation of IRE1 results in downstream signals that increase molecules involved in endoplasmic reticulum associated degradation (ERAD), targeting misfolded proteins for degradation. ATF6 activation results in the ultimate translation of more chaperone proteins to assist in protein folding. Finally, PERK activation leads to a temporary global reduction in protein synthesis by inhibiting translation (Harding et al., 2000; Hetz and Mollereau, 2014). Together, these processes allow the cell to cope with or manage accumulated misfolded proteins, and we know that these systems are activated in states of sleep deprivation. Specifically, several studies have shown that sleep deprivation leads to an increase in levels of the chaperone protein, BiP/GRP78, as well as activation of the PERK pathway of the UPR (Cirelli et al., 2004; Naidoo et al., 2005, 2007). Importantly, recovery sleep following sleep deprivation in *Drosophila* leads to a reduction in BiP levels (Naidoo et al., 2007).

Autophagy is another well-studied proteostasis pathway that is activated by the integrated stress response downstream of the UPR (see review by Kroemer et al., 2010). Autophagy leads to the degradation of aggregated proteins and defective organelles and is activated when protein homeostasis cannot be achieved by the cell, leading to apoptosis. It is known from studies in yeast that ER stress induces the autophagy response and that a downstream signaling molecule, Atg1, is necessary to induce autophagosome formation (Yorimitsu et al., 2006). In particular, it has been shown that the IRE1-JNK pathway is necessary for autophagy activation (Ogata et al., 2006). Further, ER stress molecules negatively regulate the AKT/mTOR pathway, which leads to an induction in autophagy (Qin et al., 2010). Recent data suggest that autophagy is regulated by the circadian clock with a direct role for Rev-erbα and transcription factor CEBPB linking these two processes (Huang et al., 2016). Interestingly, the cycling of autophagy proteins in hippocampal tissue is altered with sleep fragmentation (He et al., 2016). It is possible that sleep and the circadian clock could together influence how the cell is able to degrade aggregated proteins or dysfunctional organelles.

Fascinatingly, deep slow wave sleep that occurs during non-rapid eye movement (NREM) sleep, reflects the intensity of the previous waking period. The greater the previous bout of wakefulness, the longer the duration of slow wave sleep (Borbély et al., 1989). According to (Tononi and Cirelli, 2003) this slow-wave pattern could be involved in homeostatic regulation. They propose that wakefulness is associated with synaptic potentiation and slow-wave activity is associated with the corresponding synaptic downscaling, together resulting in the beneficial consequences of sleep on cognition. This hypothesis specifically addresses a role for sleep in the regulation of protein homeostasis in the brain in terms of the dynamic synaptic changes that occur with learning and daily neural activity.

## Aging: Effects on Sleep and Protein Homeostatic Regulation

The quality of sleep changes across the healthy aging process. In general, as humans reach maturity, some studies suggest that sleep need decreases (Manderville and Wetmore, 2017). Across aging, there are key changes in sleep architecture. Some of these changes include increased sleep onset latency, shorter sleep duration, impaired sleep consolidation by increased awakening, increased daytime sleepiness, decreased melatonin levels, and reduced the amount of deep slow wave sleep (Welsh et al., 1986; Pandi-Perumal et al., 2002; Wolkove et al., 2007; Yaffe et al., 2014; Mander et al., 2017 and references therein). Importantly, these age-related changes in sleep characteristics are conserved across many animal species studied (Mendelson and Bergmann, 1999; Koh et al., 2006; Naidoo et al., 2008; Wimmer et al., 2013; Brown et al., 2014). While sleep need decreases with age, the fact that sleep remains necessary even in adulthood in many species underscores that idea that sleep has been preserved across evolution because it serves a critical function.

The age-related alterations in sleep characteristics could be related to, or even contribute to, cognitive impairment. Some work has shown that poor slow-wave oscillations and sleep spindles during NREM sleep in aged individuals is linked to poor memory and forgetting (Helfrich et al., 2018). Interestingly, other work has shown that sleep quality in middle-aged individuals could affect cognitive abilities later in life. In this middle-aged population, sleep quality was positively correlated with working memory and visual-spatial episodic memory (Rana et al., 2018). Deficits in sleep at this stage could be indicative of increased risk for cognitive decline at a later age, and improving sleep quality during mid-life, could reduce susceptibility to cognitive decline. Other studies have shown that there is a correlation between poor sleep quality and working memory, long term memory, verbal knowledge, and visuospatial reasoning (Schmutte et al., 2007; Nebes et al., 2009). Further, sleep fragmentation and increased daytime sleepiness are associated with increased cognitive impairment (Ohayon and Vecchierini, 2002; Naismith et al., 2010; Jaussent et al., 2012). Importantly, sleep disruptions such as those discussed here are also associated with neurodegenerative diseases, such as AD (Ju et al., 2013; Lim et al., 2013). While the biological mechanisms and pathways that could underlie this relationship between sleep and cognition are poorly understood, it is clear that there is a connection between them with respect to aging.

Furthermore, with aging, the protein quality control systems discussed above become dysfunctional. The ER stress response becomes less adaptive as we age, resulting in reduced efficiency of the refolding aspects of the UPR and increasing the prominence of the apoptotic pathways (Hussain and Ramaiah, 2007; Naidoo et al., 2008; Brown et al., 2014). Interestingly, protein aggregations as a result of maladaptive UPR function occur in almost all of the tissues of an aged organism (see review by Koga et al., 2011). Deficits in chaperone protein function have also been reported in age-related diseases (Macario and Conway de Macario, 2002; Nuss et al., 2008), as well as overall decreases in the levels of chaperone proteins in aged wildtype rodents (Naidoo et al., 2008). Conversely, increasing chaperone protein levels results in an increase in longevity in flies and worms (Tatar et al., 1997; Kang et al., 2002; Morley and Morimoto, 2004). In addition, improving protein homeostasis by treatment with a small chemical chaperone molecule, 4-phenyl butyrate (PBA), improves sleep in aged flies (Brown et al., 2014). Further, work done in *C. elegans* has demonstrated that some protein misfolding earlier in adulthood can lead to the eventual overall collapse of protein homeostasis in older organisms (Ben-Zvi et al., 2009). It is clear that in addition to sleep quality changing with age so does overall protein homeostasis. What remains unclear is how these phenomena are related and if improving one is sufficient to rescue the other, particularly in disease states.

## Neurodegenerative Diseases: Intersection of Age-Related Changes in Sleep and Proteostasis

Protein misfolding and aggregation is a key feature of many neurodegenerative diseases, particularly AD (Cornejo and Hetz, 2013), Parkinson’s disease (PD; Breydo et al., 2012), Huntington’s disease (HD; Arrasate and Finkbeiner, 2012), fronto-temporal dementia (FTD; Ling et al., 2013), and amyotrophic lateral sclerosis (ALS; Lindberg et al., 2005). Most of those who suffer from neurodegenerative diseases are aged individuals, which is a population that, as discussed above, displays reduced sleep quality and a decreased ability to maintain protein homeostasis due to aged-related dysfunctions in cellular regulatory mechanisms [(Selkoe, 2003) and see the review by Hetz and Saxena (2017)]. Therefore, it is reasonable to consider that these aged-related changes in both sleep and protein homeostasis could increase the risk of neurodegeneration by ultimately leading to increases in levels of abnormal protein aggregates and an inability for individual cells to ameliorate this homeostatic imbalance.

The over activation of the UPR/maladaptive ER stress response could contribute to neurodegenerative disease. The expression of disease-associated proteins that aggregate and cause an imbalance in protein homeostasis have long-term consequences on the folding of other proteins. Part of the downstream signaling branches of the UPR reduces translation (Harding et al., 2000). This reduction could have negative consequences for synaptic health, memory formation, and basic cellular function, as none of these can occur without proper protein quantities. To this end, some work has shown that inhibiting PERK improves memory (see review by Halliday and Mallucci, 2014). This feature of protein regulation demonstrates that the cell does not have the capacity to deal with chronic protein misfolding, suggesting that the quality control mechanisms may be maladaptive to chronic ER stress in these disease states (Morimoto, 2008; Brown and Naidoo, 2012).

Several animal models of neurodegenerative diseases, such as ALS, FTD, AD, PD, and HD display an accumulation of misfolded proteins and an activation of the UPR (Reddy et al., 1999; Rao et al., 2002; Hoozemans et al., 2005); see review by (Hetz and Saxena, 2017). In AD, increased levels of β-amyloid precursor proteins in neurons sensitize these cells to ER stress (Chafekar et al., 2008), for review see Cornejo and Hetz (2013). Studies have shown that amyloid-β, which can accumulate in the ER lumen, can disrupt ER calcium homeostasis, leading to a proapoptotic stress response (Cornejo and Hetz, 2013). Phosphorylation of PERK and its downstream target eIF2α, a branch of the UPR, is observed in AD patients (Chang et al., 2002). Further, reduction of PERK rescues memory and cholinergic neurodegeneration by preventing increases in BACE1, which is involved in Aβ production (Devi and Ohno, 2014). Some evidence in PD has shown that ER stress in the form of increased phosphorylation of PERK and eIF2α occurs in the substantia nigra early in the disease (Hoozemans et al., 2012). In addition, another study showed that iPSCs derived from PD patients display early ER stress, which leads to a disruption of proteostasis (Chung et al., 2013). In HD, ER calcium homeostasis has been identified in patient-derived iPSCs, which could contribute to protein misfolding (Nekrasov et al., 2016), and increased levels of UPR effectors BiP and CHOP have been observed in HD patients (Carnemolla et al., 2009; Kalathur et al., 2015). Finally, all three UPR branches are expressed in ALS (Atkin et al., 2008; Hetz et al., 2009; Ito et al., 2009; Saxena et al., 2009; Kiskinis et al., 2014).

In addition, autophagy is a prominent pathological feature of many neurodegenerative diseases (see review Martinez-Vicente and Cuervo, 2007). In PD, α-synuclein can be degraded *via* autophagy (Webb et al., 2003), and it is known that the effectiveness of these degradation pathways decays with age (Martinez-Vicente et al., 2005). In AD, the lysosomal system that provides enzymes for degradation of toxic proteins in autophagosomes becomes inefficient (Yu et al., 2005). Further, in HD, macroautophagy is upregulated in response to Huntingtin aggregation (Kegel et al., 2000). It is possible that with age and poor sleep quality, increased levels of ER stress coupled with a maladaptive autophagic response all contribute to the development and progression of neurodegenerative disease.

Several studies using animal models of neurodegenerative diseases, specifically AD, have shown that increasing protein chaperone levels that assist in the folding of proteins, and thereby improve these protein quality control mechanisms, the progression of these diseases can be ameliorated (Ricobaraza et al., 2011; Wiley et al., 2011; Cuadrado-Tejedor et al., 2013; Hafycz et al., 2019). In models of ALS, treatment with eIF2α phosphatase inhibitors improved motor neuron performance and survival (Jiang et al., 2014; Wang et al., 2014; Das et al., 2015). In mouse models of FTD, PERK inhibition resulted in improved neuronal function (Radford et al., 2015). This data together suggests that reducing the activation of the UPR in these disease models can, to a degree, rescue progression of the diseases. Further evidence demonstrates that modulating ER stress signaling in an effort to make it more effective results in neuroprotection (Hughes and Mallucci, 2019). Together, these studies suggest that cellular stress underlies many neurodegenerative diseases and could serve as a therapeutic target for the treatment of patients suffering with these disorders.

As mentioned previously, most patients suffering from protein misfolding neurodegenerative diseases also report sleep disruptions (see Table 1). AD and PD in particular report a high percentage of patients suffering from sleep alterations such as increased sleep fragmentation, increased daytime sleepiness, and REM sleep disruptions (Factor et al., 1990; Iranzo, 2011; Ju et al., 2013). Additional studies examining ALS and FTD show that in a mutant animal model of these diseases expressing FUS protein demonstrate sleep and circadian disturbances prior to the cognitive deficits (Hetz and Saxena, 2017; Zhang et al., 2018). Some work has also investigated the effect of neurodegenerative disease, specifically AD, on the circadian clock, suggesting that once the neurodegeneration begins in sleep and circadian rhythm regulatory regions, the brain becomes even more susceptible to the effects of neurodegeneration (see review by Chauhan et al., 2017 and citations therein).

**Table 1:** Neurodegenerative diseases that display alterations in protein homeostatic regulation and sleep disruptions.

Disease	Evidence of ER stress in disease phenotypes	Sleep disruptions observed
Alzheimer’s Disease (AD)	Increased BiP expression in neurons of AD patients (Hoozemans et al., 2005) Increased CHOP leads to proapotosis in AD (Lee et al., 2010) Reduction of PERK rescues memory and cholinergic neurodegeneration (Devi and Ohno, 2014) Excessive eIF2α phosphorylation associated with memory loss in models of AD (Costa-Mattioli et al., 2009; Ma et al., 2013; Trinh and Klann, 2013)	Fragmented sleep, increased daytime sleepiness, REM disruptions (Prinz et al., 1982; Vitiello and Prinz, 1989; Bliwise, 2004)
Parkinson’s Disease (PD)	Neuron loss in an α-Synuclein model of PD occurs concomitantly with ER chaperone induction (Colla et al., 2012) ATF4 induction in rat dopamine neurons of the substantia nigra results in degeneration (Gully et al., 2016) ER stress in pink1/parkin models of PD leads to neurodegeneration (Celardo et al., 2016)	REM disruptions, excessive daytime sleepiness (Gagnon et al., 2002; Iranzo, 2011)
Frontotemporal Dementia (FTD)	The rTg4510 mouse model of FTD displays an increase in levels of ATF4, p-PERK, p-eIF2α, and BiP (Abisambra et al., 2013; Radford et al., 2015)	Insomnia, sleep disordered breathing, excessive daytime sleepiness (McCarter et al., 2016)
Huntington’s Disease (HD)	BiP and CHOP are upregulated in HD patient brains (Carnemolla et al., 2009) Soluble oligomers of htt activate ER stress (Leitman et al., 2013)	Increased latency to sleep, frequent nocturnal awakening, reduced sleep efficiency (Wiegand et al., 1991; Morton et al., 2005)
Amyotrophic Lateral Sclerosis (ALS)	Elevated levels of ER stress markers, CHOP, XBP1s, and BiP/GRP78 in motor neurons in an animal model of ALS (Ito et al., 2009; Wang et al., 2011; Das et al., 2015)	Daytime sleepiness, sleep disordered breathing (Ferguson et al., 1996; Lo Coco et al., 2011)

An ongoing investigation into these diseases is required to establish which comes first, protein homeostatic disruptions or sleep disturbances, and in particular, if sleep quality is improved, can the spread of these protein misfolding diseases be delayed or halted altogether?

## Concluding Remarks

There is sufficient evidence that cellular health and sleep quality go hand-in-hand. Across the aging process, both sleep quality and protein homeostatic mechanisms decrease in efficiency. Many studies demonstrate that poor sleep quality is correlated with poor memory and cognition. While it is impossible to examine protein homeostasis and protein levels of neurons in humans, evidence from neurodegenerative diseases clearly shows increases in cellular stress and poor regulation of protein folding, leading to aggregations commonly seen in these diseases. What remains unclear is the link between sleep and protein homeostasis in these disease states. Some evidence suggests that it is the interplay between poor sleep and maladaptive protein regulation that contributes to age-related neurodegenerative diseases like AD. We suggest, since both sleep quality and the efficacy of protein homeostatic maintenance machinery decreases with aging, it seems plausible that these two key aspects of aging could contribute to the onset of neurodegenerative age-related diseases. In accordance with this perspective, improving sleep quality and relieving cellular stress could serve as critical target areas for therapeutic intervention of these devastating diseases.

## Author Contributions

JH and NN wrote and edited the manuscript.

## Conflict of Interest Statement

The authors declare that the research was conducted in the absence of any commercial or financial relationships that could be construed as a potential conflict of interest.
